# Writing with Molecules: Tip-Induced Local Chemisorption of N‑Heterocyclic Olefins on Cu(111)

**DOI:** 10.1021/jacs.5c06188

**Published:** 2025-07-23

**Authors:** Felix Landwehr, Ankita Das, Sergio Tosoni, Juan J. Navarro, Mowpriya Das, Frank Glorius, Markus Heyde, Beatriz Roldan Cuenya

**Affiliations:** † Department of Interface Science, 28259 Fritz-Haber Institute of the Max-Planck Society, 14195 Berlin, Germany; ‡ Universität Münster, Organisch-Chemisches Institut, 48149 Münster, Germany; § Dipartimento di Scienza dei Materiali, 9305 Università di Milano-Bicocca, Via Cozzi 55, 20125 Milano, Italy

## Abstract

N-Heterocyclic olefins (NHOs), possessing highly polarizable electron-rich double bonds, have recently received increased attention as promising ligands to modify the properties of various surfaces such as Cu, Au, and Si. This work demonstrates the precise “writing” of molecules on a Cu(111) surface by using the electric field of a scanning tunneling microscope (STM) tip. This selectively switches the molecules from a mobile, physisorbed state to a chemisorbed state with molecular, nanoscale spatial resolution under ultrahigh vacuum conditions. We utilize STM and high-resolution electron energy-loss spectroscopy supported by density-functional theory to investigate adsorption and orientation of the molecules on the Cu(111) surface. We find that IPr-NHO adopts two distinct adsorption states on Cu(111): a mobile, physisorbed state and a chemisorbed state. We can distinguish between the two states using X-ray photoelectron spectroscopy and show that the mobile species can be transformed into the immobile species via interaction with the STM tip with molecular precision. This enables “writing” of IPr-NHO on Cu(111) as the molecules can be chemisorbed in a predefined assembly in intentionally designed shapes, opening new possibilities for nanofabrication, molecular electronics, and tunable surface chemistry.

## Graphical abstract

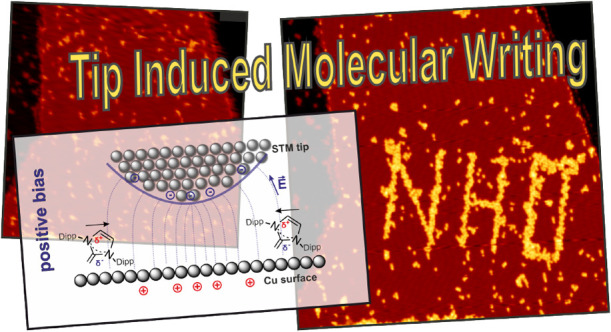

## Introdution

Surface modification is not only of fundamental interest but also highly relevant for applications in the fields of heterogeneous catalysis,^1^ microelectronics^2^ or sensing.^3^ A commonly employed strategy for larger scale modification are self-assembled monolayers (SAMs), where organic molecules act as building blocks of spontaneously assembled structures.^4^ However, if molecular assemblies can be controlled by external stimuli this opens the door for creating molecular assemblies on a nanometer scale.^5^

On the scale of individual or very few atoms and molecules, STM tips can be used to exert different types of forces on surface species for controlled manipulation. In their pioneering STM work, Eigler and Schweizer^6^ demonstrated the controlled alignment of individual Xe atoms on Ni(110) at liquid helium temperatures. This marked the first ever demonstration of using an STM tip to manipulate individual atoms. Using the forces between the tip, surface species and the surface itself allows for control of individual atoms and molecules with even intramolecular precision and enables switchable molecules and molecular rotors.^7–9^ Another way of manipulating surface species is by utilizing the electron tunneling current itself. Vibrations can be excited in molecules by inelastically tunneling electrons, which in turn can induce various surface reactions.^7^

Additionally, the electric field generated between the STM tip and the surface provides a powerful means of controlling adsorbed species, particularly those possessing a charge or dipole moment. Studies on this topic have utilized physisorbed molecules stabilized by dipole interactions, both between the molecule and the surface^10^ and among assembled molecules.^11^ Additionally, the conformational changes of adsorbed molecules have been explored.^12^

Promising ligands to form SAMs on metals, metal-oxides and semimetals are N-heterocyclic carbenes (NHCs).^1,13–40^ NHC-based SAMS have been shown to exhibit chemical and thermal stability^1,17,18,37,41,42^ as well as versatility when it comes to molecular design.^19,33,43,44^ This stems from their ability to covalently bind to a variety of surfaces via σ-bonding and π-backbonding.^13,18,23,36^ Among the several surface science techniques to investigate NHCs, vibrational methods such as tip-enhanced Raman spectroscopy or high-resolution electron energy-loss spectroscopy (HREELS) have been utilized as powerful tools to investigate binding modes and adsorption geometries.^17,19,45–50^

Recently, N-heterocyclic olefins (NHOs) have emerged as viable class of molecules for the functionalization of metals and semiconductors.^48,51,52^ They are structurally derived from NHCs through the introduction of an additional methylidene moiety.^53,54^ This introduces an electron-rich, and highly polarizable double bond which leads to a strong nucleophilicity based on the ylidic character of the double bond.^55^ In this work, we present the functionalization of a Cu(111) surface by a diisopropylphenyl substituted NHO (IPr-NHO). The corresponding IPr-NHC has been reported by the authors on Cu(111), Cu(100) and oxidized Cu(111) surfaces.^25,26,56^ Here, we provide insight into the binding geometry and adsorption configuration of IPr-NHO. Moreover, we found that IPr-NHO can be locally transformed to a chemisorbed configuration on Cu(111) using the electric field of an STM scanning tip on a nanometer scale.

## Experimental Section

IPr-NHO was introduced into the UHV system via its IPr-NHO–CO_2_ adduct precursor, synthesized following a reported procedure.^52^ The Cu(111) surface was prepared by consecutive cycles of Ar^+^-ion bombardment at 1 kV and annealing at 920 K. IPr-NHO molecules were deposited by resistive heating of the corresponding CO_2_-adduct at an evaporation temperature of 320 K with the sample kept below 310 K. C 1s, O 1s, Cu 2p and Cu LMM XPS measurements confirmed the clean deposition of the decarboxylated, free NHO on the clean Cu(111) surface (Figure S10).

## Results and Discussion

To investigate the adsorption behavior of IPr-NHO, we utilized low-temperature (5 K) STM measurements. At low coverage (Figure 1a), the molecules adsorb individually, occasionally forming dimers and even aggregations of multiple molecules without apparent ordering. The molecules display an almost rectangular shape (Figure S1) which could indicate limited rotation induced by the tip movement.

**Figure 1:**
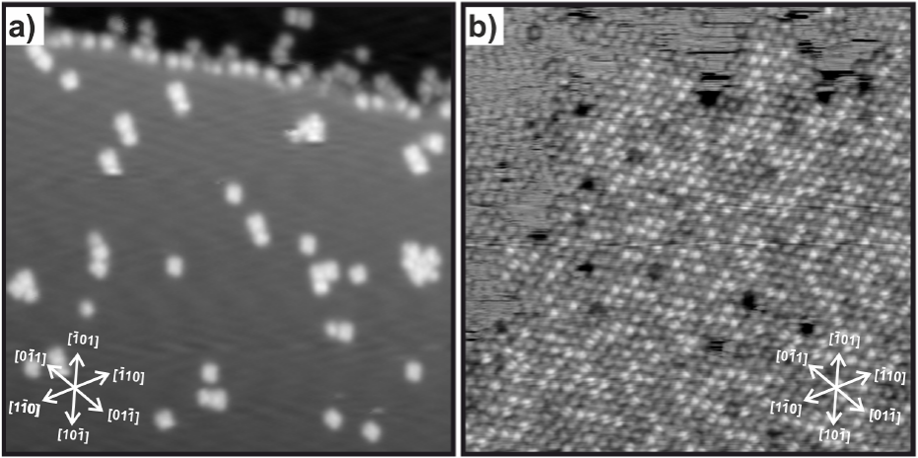
IPr-NHO molecules on Cu(111). (a) Coverage of 0.03 ± 0.01 nm^–2^. 40 nm × 40 nm, *V*_s_ = −0.8 V, *I*_t_ = 9 pA. (b) Coverage of 0.67 ± 0.09 nm^–2^. 40 nm × 40 nm, *V*_s_ = −1.3 V, *I*_t_ = 15 pA.

At higher coverage (Figure 1b), IPr-NHO forms large molecular domains which arrange in a close packed hexagonal lattice with a molecule–molecule distance of 1.1 nm. At full coverage (Figure S2), the molecules form a continuous film with a close packed hexagonal lattice. On all images taken for full coverage, the orientation was aligned with high-symmetry directions of the underlying Cu(111) substrate. In Wood notation the molecular lattice can approximately be described with $\left(\sqrt{19}\times \sqrt{19}\right)$ .

The binding orientation of IPr-NHO was further investigated via HREELS. In Figure 2 the HREELS spectra of IPr-NHO is compared to those of IPr-NHC, for which the adsorption geometry displayed in Figure 2c was found by DFT and experimentally by STM.^25^ Here, we utilize HREELS to obtain the spectrum in Figure 2a for comparison with the spectra of IPr-NHO. HREELS measurements were conducted at a sample temperature of 80 K.

**Figure 2:**
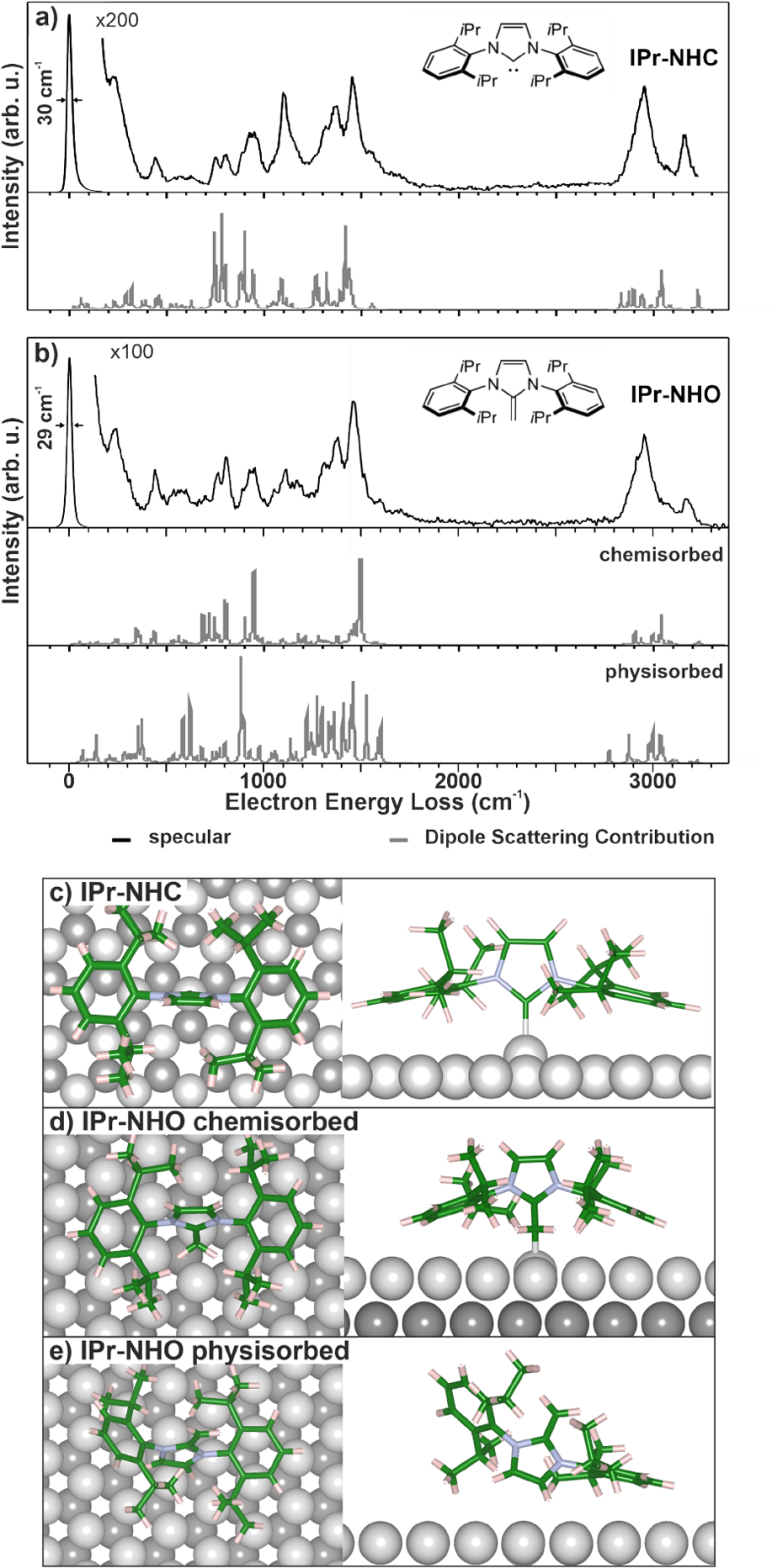
Binding geometry of IPr-NHO and IPr-NHC. Vibrational HREELS spectra were measured in specular scattering geometry of submonolayer coverages of (a) IPr-NHC and (b) IPr-NHO together with DFT-calculated intensities and frequencies of the optimized binding geometries produced by the dipole scattering (gray) mechanism. The fwhm (full-width at half-maximum) of the elastic scattering peak is given as a measurement of resolution. The DFT-optimized binding geometries of (c) IPr-NHC, (d) IPr-NHO (chemisorbed), and (e) IPr-NHO (physisorbed) in top-view (left) and side-view (right) are also displayed. Figure 2c has been adapted with permission from previous work of this group^25^ available under a CC BY-NC-ND 4.0 license. Copyright 2022 Navarro.

The signals between 500 and 1500 cm^–1^ can be divided into four signal groups. Comparison with other HREELS works of NHCs^17,19,48–50^ as well as our DFT calculations (Figure S13) was utilized for the interpretation of the spectra. Centered around 780 cm^–1^, C–C bending vibrations as well as aromatic C–H bending vibrations can be found. Both molecules display two identifiable peaks around 760 and 800 cm^–1^. These are associated with aromatic and alkylic C–C bending vibrational modes. An additional broad signal is observed around 920 cm^–1^, followed by a strong signal at 1100 cm^–1^ associated with C–H bending vibrations. Finally, a broad signal consisting of multiple peaks is observed between 1300 and 1500 cm^–1^. These signals are associated with C–C stretching vibrations typically found in aromatic systems. In addition, at the high energy end, the signals at 2950 and 3160 cm^–1^ are associated with alkyl and aromatic C–H stretching vibrations, respectively. The spectrum for IPr-NHC is plotted together with the DFT calculated vibrational modes that significantly contribute to the dipole scattering of the electron beam in HREELS based on the adsorption geometry shown in Figure 2c. These vibrational modes contain a dynamic dipole moment perpendicular to the surface, leading to an increased signal strength and typically dominate HREELS spectra. The DFT calculated peaks are in good agreement with the experimental spectrum. An assignment of the DFT calculated peaks can found in Figure S13.

When considering the HREELS spectra, both spectra show very similar features, indicating similar adsorption geometries. Most notably, IPr-NHO shows a less pronounced signal at 3160 cm^–1^ as well as around 1100 cm^–1^. These signals were attributed to aromatic C–H stretching vibrations and the aromatic backbone vibrations of the central ring. The lower intensity for IPr-NHO indicates that the imidazole ring of IPr-NHO is tilted with respect to the surface, while for IPr-NHC the backbone is normal to the surface. DFT calculations find a tilt of 50.8° for the IPr-NHO backbone in the binding geometry shown in Figure 2d.

Noteworthy for both molecules is the vibration at 440 cm^–1^. Modes in this range have previously been associated with C–Cu bonds. This indicates that both molecules chemisorb to the surface. The similar vibrational energy also highlights the similar bond length, which we found to be 2.0 Å for IPr-NHC^25^ and 2.1 Å for IPr-NHO by DFT.

However, while the similarity between the HREELS spectra of IPr-NHO and IPr-NHC reveals a similar binding geometry, the DFT calculated spectrum for IPr-NHO predicts some differences between the dipole-active modes of the two adsorbed molecules due to the different angles of the aromatic rings. We associate the differences between theory and experiment with the presence of physisorbed species of IPr-NHO on the surface. Particularly a small feature at 1590 cm^–1^ indicates the presence of an olefinic feature. The DFT calculated spectrum is based on the physisorbed adsorption geometry shown in Figure 2e.

A method routinely employed in STM studies is bias dependent measurements. For IPr-NHO on Cu(111), it proved challenging to obtain satisfactory images at positive bias (Figure S3). The images taken at small positive biases are very fuzzy even at lower coverages which could be related to tip-induced molecule mobility. Therefore, most LT-STM measurements in this work were performed at a negative bias. However, when scanning an area at negative bias after scanning the same region at larger positive bias (typically >2.3 V), additional molecules appeared. Figure 3a shows an image taken at negative bias, on which a smaller image was successively measured at positive bias (Figure 3b). The same area was then remeasured at the same initial measurement conditions (Figure 3c). The change is quite drastic, as the whole area exposed to a positive tip bias is covered by molecules. The 2D FFT insets in Figure 3a,c indicate that the molecules do not follow any particular order, as only a diffuse ring can be observed, indicating the smallest possible distance between molecules without any specific organization. Instead of scanning an image at positive bias, the same effect can be achieved when the tip is guided across the surface while a positive bias is applied. By carefully switching between positive and negative bias, complex structures only a few molecules wide can be created, as shown in Figure 3d. Moreover, for Figure 3e a total of 9 rectangular areas where scanned twice each successively at positive bias. This results in the emergence of new molecules at an even larger scale, incorporating a large amount of previously not observed molecules. Between scanning each individual segment of the structure the bias of the STM tip had to be switched back to a negative bias to avoid the “writing” of molecules when switching between each letter. This indicates the stability of the “written” molecules against changes in the tip–sample bias. It is important to note that molecules observed before the “writing” process in the vicinity of the process are not affected by that process, as indicated by the marked molecules in Figure 3a,c. When measuring at moderate to low positive bias, some additional molecules can sometimes be observed compared to images measured previously at negative bias. Moreover, measuring at these conditions also results in additional molecules in subsequent images measured again at negative bias (Figure S8). The “written” molecules in Figure 3d appear at an apparent height of 241 ± 16 pm while the previously present molecules appear at the same apparent height of 231 ± 16 pm.

**Figure 3:**
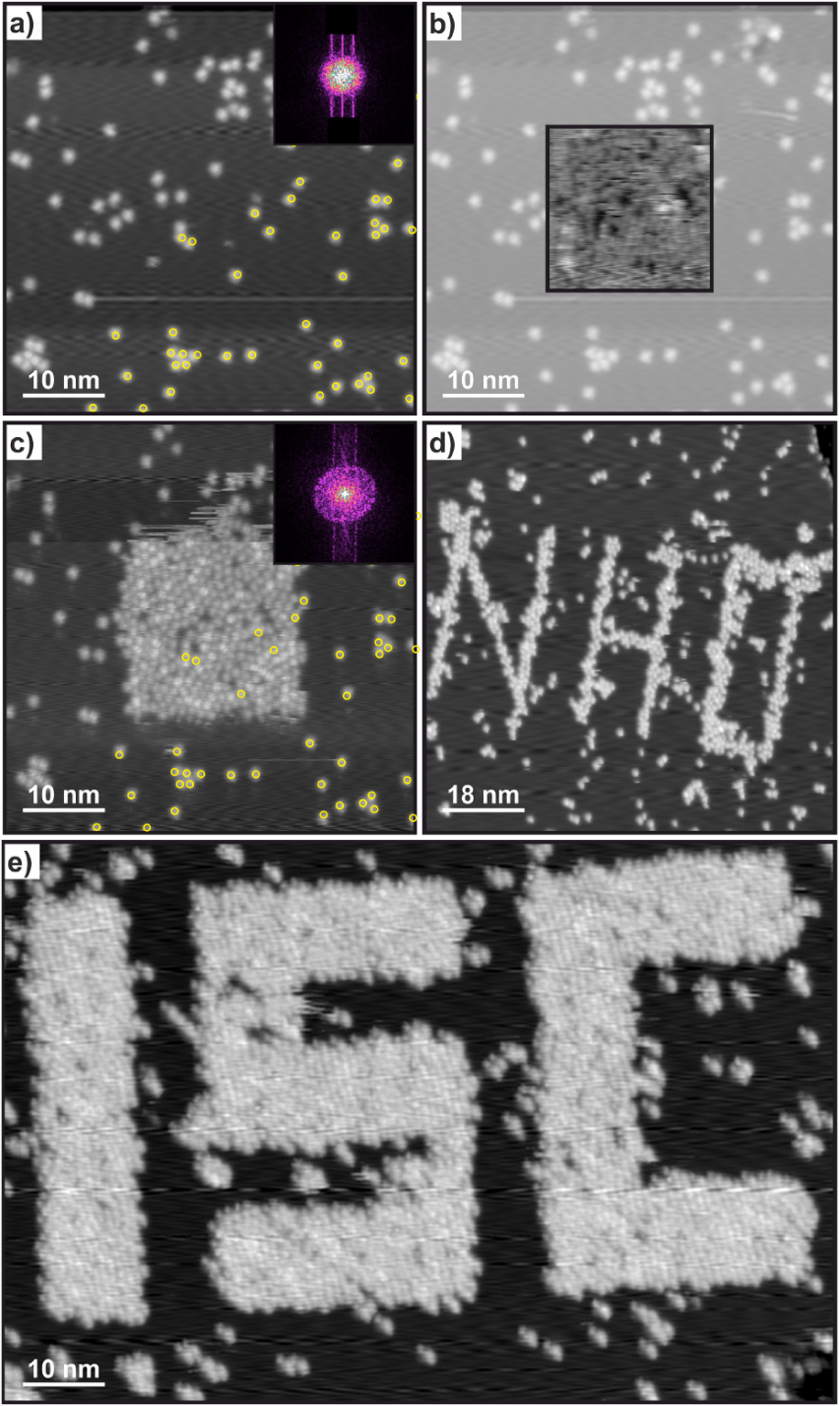
IPr-NHO molecules on Cu(111) showing deposition after molecular “writing” conditions. (a) 50 nm × 50 nm, *V*_s_ = −1.3 V, *I*_t_ = 9 pA image of individually adsorbed IPr-NHO molecules. (b) Overlay of an image recorded at a positive scanning bias. 20 nm × 20 nm, *V*_s_ = +2.4 V, *I*_t_ = 20 pA. (c) Subsequently scanned image at the same conditions as recorded for (a). (d) Molecules “written” by guiding the STM tip over the surface at +2.6 V and 9 pA. Image conditions: 80 nm × 80 nm, *V*_s_ = −0.8 V, *I*_t_ = 9 pA. (e) Molecules “written” by scanning in different rectangular scan frames at +2.6 V and 20 pA. Image conditions: 101 nm × 68 nm, *V*_s_ = −0.8 V, *I*_t_ = 9 pA. The insets in (a,c) are the 2D-Fast-Fourier-Transform of the respective images at a size of 4.6 nm^–1^ × 4.6 nm^–1^. Some molecule positions have been marked in a and c, showing no displacement of molecules after the “writing” process.

A way to manipulate particles on a surface is by picking up that particle with the STM tip, and later putting it down on the surface.^7^ This would result in a mechanism similar to dip-pen nanolithography (DPN), however without the need of water or any other solvent as a mediator.^57^

This mechanism was tested by first building a “mobility cage” on the surface by creating the outline of a square out of molecules by the previously described process (Figure 4a,b). Subsequently, a rectangular image was measured at “writing” conditions over the boundary of the cage (Figure 4c). Half of that rectangle lies within the created square, while half of it is outside. As a result, the scanning area outside the mobility cage is fully covered in molecules, while inside the cage only a part of the area is covered. If the molecules were manipulated vertically in a mechanism similar to DPN, on-surface obstructions should not influence the molecular deposition. Rather, this observation strongly indicates that there seems to be a finite amount of molecules that can be “written” in a given space. These molecules are not only located in the scanning area but must be attracted toward the tip. Moreover, these molecules have to be able to move laterally toward the area under the tip. This observation is critical to the explanation of the observed effect. It represents direct experimental evidence of the on-surface movement of IPr-NHO being confined in a two-dimensional space.

**Figure 4:**
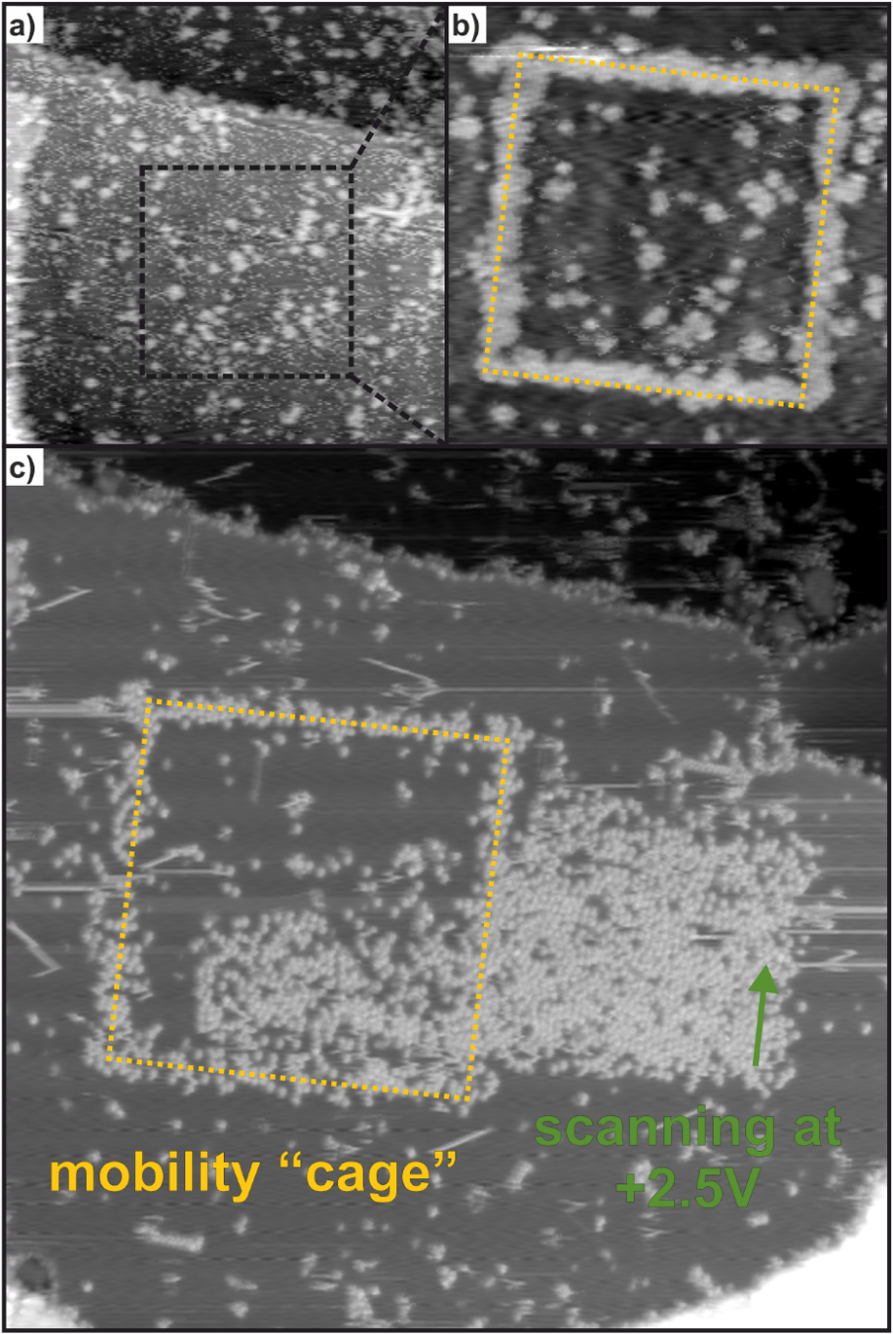
STM images tracking the “writing” of IPr-NHO/Cu(111). All images were measured at *V*_s_ = −0.9 V, *I*_t_ = 9 pA. (a) 120 nm × 120 nm. The area before a writing attempt. (b) 70 nm × 70 nm. A square is “written” on the surface by dragging the tip along the marked path two times at a speed of 8 nm/s while holding *V s* at +2.5 V and *T*_*t*_ at 150 pA. This creates a physical barrier for translational movements of molecules. (c) 120 nm × 120 nm. Previous to this measurement, a rectangular STM scan was taken in the area marked in green (80 nm × 35 nm, *V*_s_ = −0.9 V, *I*_t_ = 150 pA) from bottom to top, indicated by the green arrow.

A similar experiment across a Cu step-edge indicates that the same holds true for terrace boundaries with molecule “writing” not uniformly over step-edges (Figure S5). This experiment highlights that at the sample conditions in this setup, molecules are confined to their terraces and cannot traverse laterally over the step-edges. These experiments indicate molecules need to move laterally over the surface before they are “written.” As all STM measurements were performed at a sample temperature of 5 K, thermal diffusion can be excluded, implying that any movement of the molecules is directionally induced by interaction with the tip. This implies two species on the surface: a species readily observable at negative scanning conditions as well as a second, more mobile species. At higher negative bias these species cannot be observed, indicating a repulsive force of the tip, while at positive scanning bias the latter species is attracted by the tip. As shown in Figure S6, the “written” molecules remain in place after scanning at negative bias for 10 h, indicating a similar kinetic barrier than the initially observed species on the surface. The effectiveness of the “writing” process can also be influenced by the tip–sample-distance. A higher tip–sample distance leads to a lower yield of the writing process under the same bias conditions and tip speed (Figure S7).

Incidentally, the low-intensity, broad N 1s XPS spectra (Figure 5a) can also be separated into two different components. The positions were verified through multiple measurements due to the poor signal-to-noise-ratio. The first component is located at a high binding energy of 403.0 ± 0.2 eV, the second component at 401.4 ± 0.3 eV. These signals may be related to the chemisorbed and the mobile, physisorbed species, respectively. The first signal is consistent with previously reported IPr-NHO on Si(111) (402.7 eV),^52^ where a strong charge transfer of the molecule upon binding to the surface has been observed. The second peak is at a similar position as the N 1s signal of the structurally similar IPr-NHC which we have discussed on Cu(111) in a previous publication (401.1 eV).^26^ This highlights the inherent intramolecular polarization of IPr-NHO even when physisorbed, which leads to a binding energy comparable to chemisorbed IPr-NHC, where the electron density in the ring has already been decreased due to the binding. XPS spectra were taken at room temperature, which indicates the two species observed in STM at 5 K are present still at room temperature.

**Figure 5:**
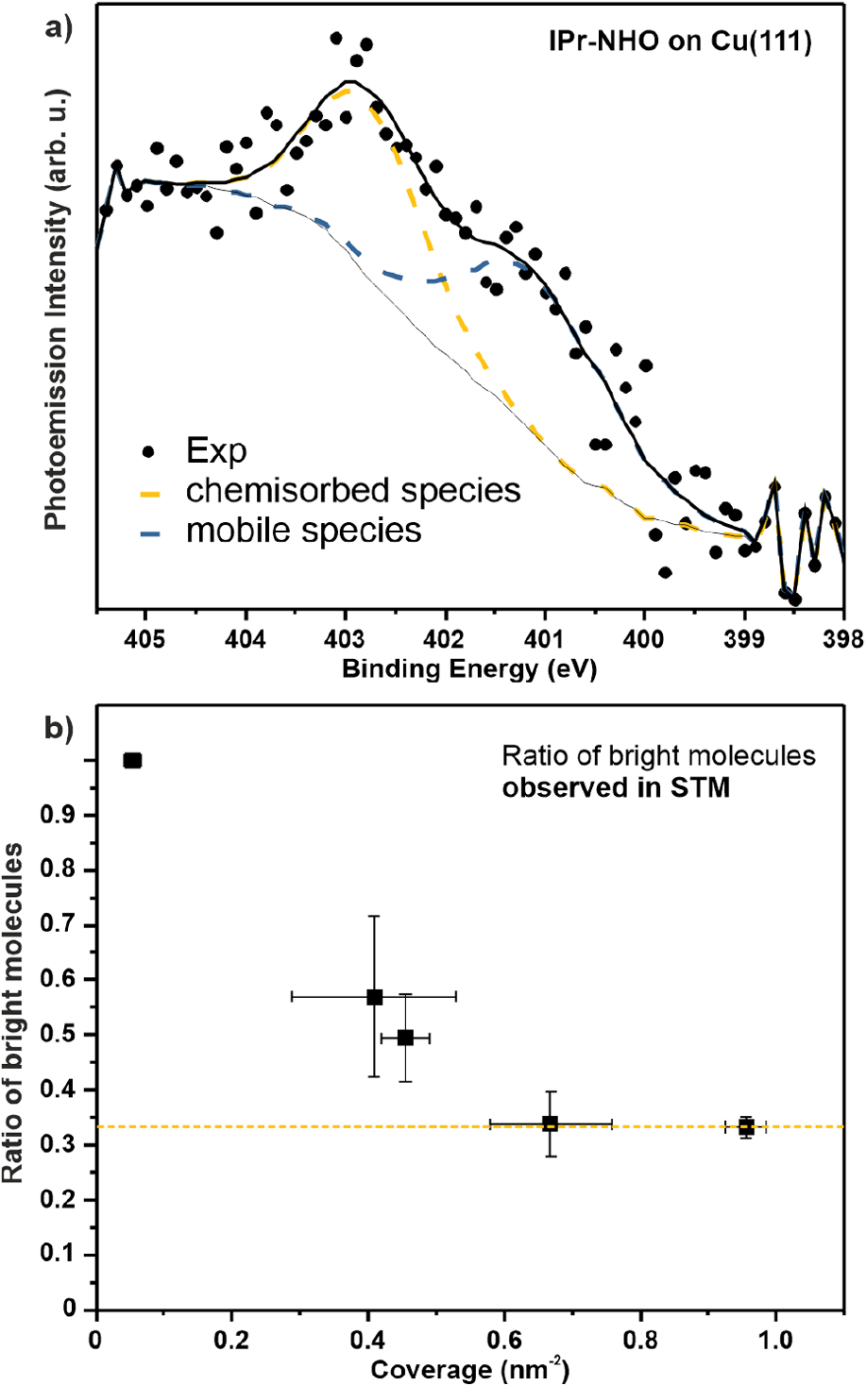
Observation of chemisorbed and mobile species on Cu(111). (a) N 1s XPS spectrum of IPr-NHO. The spectrum is representative of multiple spectra taken for several sample preparations and coverages. From these spectra, the positions of the two components were found at 403.0 ± 0.2 and 401.4 ± 0.3 eV. (b) Fraction of molecules appearing at a higher apparent heights in the STM measurements as a function of their total coverage of the molecules. At low coverage, the molecules appear at a uniform apparent height, leading to a ratio of 1.0. At full coverage, the “bright” species are present at a ratio of 0.33 ± 0.02.

The presence of two distinct species of IPr-NHO is also in agreement with STM measurements. At first glance in Figure 1b, the observed molecules do not appear uniformly, but at different apparent heights. They can be broadly categorized into “bright” and “dark” species. In the periphery of the molecular domains, molecules appear darker. Moreover, the streaks at the domains edge indicate a high degree of molecular mobility in this region, preventing clean STM imaging. Given that the measurements were conducted at a sample temperature of 5 K, thermal diffusion can be ruled out, suggesting that a fraction of the molecules adopt an adsorption geometry that allows easy displacement due to tip interactions. The height difference between bright and dark molecules was found to be between 20 and 60 pm, depending on imaging and tip conditions. However, at closer inspection the distribution between these species changes with molecule coverage. At low coverage, only one species can be observed. With increasing coverage, the fraction of brighter species decreases relative to the total molecules. At full coverage of 0.96 ± 0,03 nm^–2^, the bright species make up 33 ± 2% of all molecules (Figure 5b). Moreover, the darker species seems to be more mobile as observed by the difficulty of imaging these molecules at the edges of molecular domains.

We hypothesize, that IPr-NHO adsorbs on the Cu(111) surface in two distinct configurations: as a chemisorbed species, bound directly to the surface as shown in Figure 2c, and as a physisorbed species with a low translational barrier. The HREELS spectra supports the presence of additional species besides the chemisorbed species. At low coverages, the physisorbed species are easily moved by the STM tip at a negative bias, leaving us unable to image them. With increasing coverage, the increasing amount of chemisorbed species will obstruct the movement of the physisorbed species, making a fraction of them observable by STM. At full coverage all molecules are sterically fixed in position, making it possible to observe all physisorbed species as “dark” molecules (see Figure 1b within the patch). To support this hypothesis, IPr-NHO was evaporated on a Cu(100) surface. Previous work with IPr-NHC on this facet has revealed a stronger binding energy of IPr-NHC on Cu(100) as compared to Cu(111).^25^ The STM images are found in Figure S9. The molecules were evaporated at conditions that lead to an apparent coverage of 0.06 ± 0.01 nm^–2^ on Cu(111). Interestingly, on Cu(100) a coverage of 0.19 ± 0.13 nm^–2^ was obtained, while the “writing” process was not observed. This indicates all molecules bind to Cu(100), preventing the formation of a mobile species.

DFT calculations indicate that IPr-NHO molecules can adsorb on Cu(111) in two different modes. Nonspecific physisorption (Figure S11) is dominated by dispersive interactions. The exo-C atom maintains its sp^2^ hybridization, as shown by the C–C distance (1.36 Å) and the HCH angle (120.4°). The adsorption energy is −2.65 eV. A more stable chemisorbed structure is obtained if the exo-C undergoes an sp^2^ to sp^3^ transition, and a new C–Cu bond is formed (Figure S11). The C–Cu bond distance is 2.11 Å, and the change in the C hybridization is proven by the elongation of the C–C bond (1.42 Å) and the closing of the HCH angle to 110.4°. The adsorption energy is larger, −3.21 eV. The physisorbed molecule, given the absence of direct chemical bonds to the surface, is supposedly highly mobile. The presence of a strong C–Cu bond, on the contrary, anchors the chemisorbed species to the surface. The calculated BE (N 1s) supports the interpretation of the XPS spectra discussed above, since a binding energy of 379.6 eV is assigned to the chemisorbed species, while a smaller BE (378.7 eV) is obtained for physisorbed NHOs.

To explain the observed behavior of IPr-NHO on the surface, the molecule’s inherent dipole moment μ must be considered. We derive from DFT calculations, that both, the physisorbed species as well as the chemisorbed species, contain a dipole moment perpendicular to the surface directed from the molecule to the surface of −1.25 |*e*|Å and −2.13 |*e*|Å, respectively (Figure 6a,d). Moreover, it is important to recall that the STM tip above the surface creates a strong electric field (typically *E* ≥ 10^7^–10^8^ V cm^–1^ at a tip–sample-distance of 1 nm). Along the surface, the field is nonuniform and increases in intensity under the tip apex. The electric field of the tip has been used to manipulate molecules and atoms on the surface, often carried out as pulses.^7,11,12,58^ The effective range of these manipulations has been reported at over 100 nm around the tip. The movement of individual molecules across a surface due to attractive and repulsive electric field forces has been observed, for example, for dibromoterfluorene on Ag(111).^59^ The manipulation of chemical reactions via electric field has also been shown for example for Diels–Alder reactions.^60^ By stabilizing the intramolecular charge separation toward one particular resonance (similar to our proposed mechanism) the bond-forming process can be accelerated. More generally, the influence of electric field along the reaction-axis or direction of electron reorganization has been shown to influence a number of chemical reactions at will, leading the way toward smart reagents via so-called oriented external electric field (OEEFs).^61^

**Figure 6:**
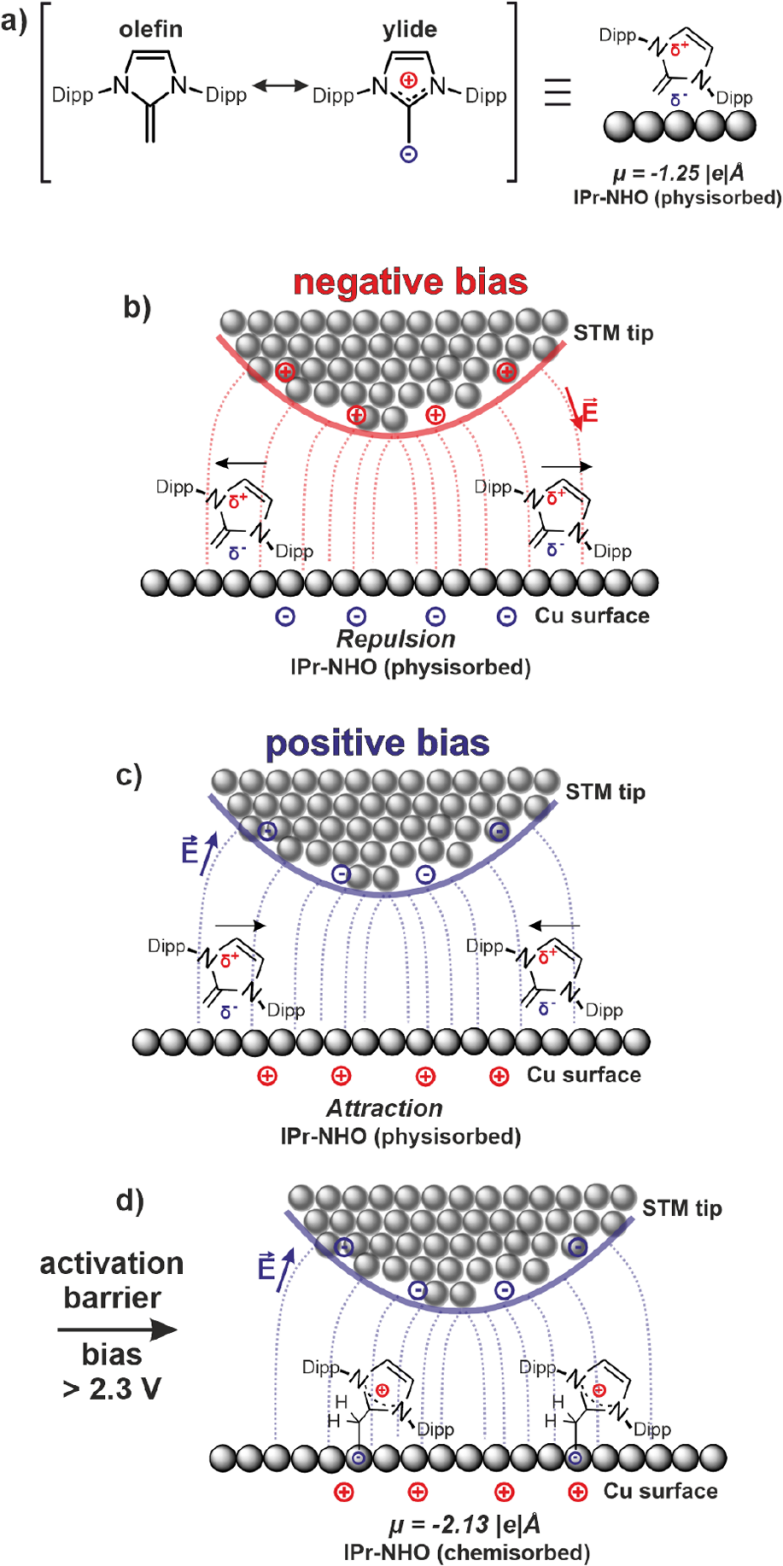
Graphic illustration of the proposed field-determined writing mechanism. (a) Dipole moments of the physisorbed IPr-NHO molecules are based on the ylidic resonance structure and are indicated schematically by partial charges. (b) Negative electric field is defined as pointing toward the surface. Repulsive behavior in a negative electric field. (c) Attractive behavior in a positive electric field. (d) At a sample voltage exceeding 2.3 V, the physisorbed molecules are transformed into chemisorbed species in an electric field created by a positive bias. The dipole moments μ were determined from DFT calculations.

Due to the orientation of the dipole moment μ of IPr-NHO on the surface, the tip will exert a potential wall of μ × *E* on the molecule when at a negative bias and therefore act repulsively toward the molecules’ dipole moment perpendicular to the surface (Figure 6b). Therefore, the physisorbed species will be pushed out of the tunneling junction and therefore cannot be imaged by STM at negative bias. Conversely, at a positive bias the electric field represents a potential well, leading to attractive forces on the molecules’ dipole moment (Figure 6c). This leads to molecules being attracted to the tunneling junction, leading to noisy imaging conditions as seen in Figure S3.

This effect can also explain how the molecules are fixed once under the tip at sufficiently large positive bias. DFT calculations show that the relative stability of the chemisorbed species over the physisorbed species increases in an increasingly positive external electric field (Figure S12) as expected for the stronger negative dipole moment of the chemisorbed species. If the transition from the physisorbed species to the chemisorbed species is an activated process it would involve flipping the central heterocycle relative to the IPr side groups, as well as the transition of the exocarbon from an sp^2^ to a sp^3^ hybridization. The potential energy gain within the electric field under the apex could help the molecule over the activation barrier, leading to chemisorption. This is schematically indicated in Figure 6. This “writing” effect should be considered a mechanism generally valid for molecules on the surface. However, one can expect this mechanism will be governed by the precise interplay of different adsorbate properties. First, a somewhat mobile species with a dipole moment perpendicular to the surface needs to exist. Second, the barrier for lateral movement needs to be low enough that the electric field generated by the STM tip can either attract or repulse this species. Finally, a less-mobile configuration needs to exist. This species either needs to contain a stronger dipole moment perpendicular to the surface than the original species or it needs to be reached via a transition state that can be stabilized via the electric field. Our experiments on Cu(100) show that changing the substrate facet can already alter the conditions on the surface enough for this process to be prevented. Therefor, for other surfaces such as Ag, Si, Au or metal oxides, an adsorbate that fulfills the mentioned conditions could be envisioned but must be tailored to the specific surface to achieve such a “writing” process.

Another possible mechanism for the “writing” process involves inelastic tunneling (IET) of the tunneling electrons. IET can induce vibrational, electronic or vibronic excitations in adsorbates, which could provide the energy necessary to induce surface reactions and chemisorb the molecules to the surface.^7,62^

## Conclusions

In conclusion, IPr-NHO adsorbs on Cu(111) in two distinct states, which we identify as a physisorbed and a chemisorbed species. The physisorbed species exhibits a high degree of repulsive mobility induced by the electric field when measured under negative bias conditions, making the observation of this species by STM difficult. However, at positive bias, the effect reverses and the molecules are attracted toward the tip. Moreover, at close proximity to the tip the mobile species gets converted into a less mobile, chemisorbed species. These effects enable the “writing” of molecules on the surface by dragging the tip over the surface at a positive bias in intentionally designed shapes.

## Supplementary Material



## Notes

Methods, thermal stability measurements of IPr-NHO/Cu(111) via STM and HREELS, STM of “written” structures over extended timelines, MS data of IPr-NHO, additional STM images of IPr-NHO at different STM bias, XPS: Cu 2p, CuLMM, and C 1s peaks, and DFT calculations for simulated HREELS spectra (PDF)
