# Supplementary material for: Writing with Molecules: Tip-Induced Local Chemisorption of N‑Heterocyclic Olefins on Cu(111)
Source: J Am Chem Soc. 2025 Jul 23;147(31):27676–84. doi: 10.1021/jacs.5c06188 (PMC12333018; doi:10.1021/jacs.5c06188)
Supplement: Supplementary file 1 [file ja5c06188_si_001.pdf]

Supporting information

Writing with Molecules: Tip-Induced Local Chemisorption of N-Heterocyclic Olefins on Cu(111)

Felix Landwehr,[†] Ankita Das,[‡] Sergio Tosoni,[*,¶] Juan J. Navarro,[†] Mowpriya Das,[‡] Frank Glorius,[*,‡] Markus Heyde,[*,†] and Beatriz Roldan Cuenya[†]

† Department of Interface Science, Fritz-Haber Institute of the Max-Planck Society, 14195 Berlin, Germany

‡ Universität Münster, Organisch-Chemisches Institut, 48149 Münster, Germany

¶ Dipartimento di Scienza dei Materiali, Università di Milano-Bicocca, Via Cozzi 55, 20125 Milano, Italy

Corresponding authors:

[*]ST, sergio.tosoni@unimib.it; [*]FG, glorius@uni-muenster.de;
[*]MH, heyde@fhi.mpg.de

Contents

Contents 1
A.- Methods 2
B.- IPr-NHO on Cu(111) 4
C.- “Writing” Process of IPr-NHO on Cu(111) 7
D.- IPr-NHO on Cu(100) 11
E.- XPS 12
F.- Calculated Structures HREELS Vibrational assignment 13
G.- References 16

A.- Methods

The synthesis of 1,3-bis(2,6-diisopropylphenyl)-2-methylene-2,3-dihydro-1H-imidazol-3-ium-2-carboxylate (IPr-NHO-CO_2 adduct) was carried out as described in previous publications.[1] The ligands were deposited on the substrate under UHV conditions (~ 5 $\times 10^{-10}$ mbar). The IPr-NHO-CO_2 adduct was heated in a Knudsen cell (Kentax) at 320 K, resulting in the generation of the free IPr-NHO with only CO_2 as a by-product.

Cycles of sputtering with Ar^+ at 1 kV for 30 minutes and annealing at 950 K for 5 minutes were employed to prepare a clean Cu(111) surface.
The STM images were taken at 5 K under pressures < 2 $\times 10^{-10}$ mbar using a PtIr tip, in constant current mode. In the main text, V_s denotes the bias voltage at the sample and I_t the tunneling current.

XPS measurements were performed using a SPECS non-monochromatic X-ray source with an Al anode (E_{Photon}: 1486.61 eV) operating at 300 W and a Phoibos100 analyser. The background of the spectra was subtracted through the Shirley method. For the fittings, Gaussian-Lorentzian functions were used. For the deconvolution of the C 1s spectra, the appropriate ratio of chemically different carbon atoms was employed as an area constraint while keeping the FWHM under 2.0 eV.

All high-resolution electron energy loss spectroscopy (HREELS) spectra were acquired at T < 80 K using an incident beam energy of 3.5 eV. For the spectra obtained in the specular geometry, the analyser angle was set at 67° with respect to the surface normal. For the off-specular measurements, the analyser was moved until the intensity of the elastic peak dropped to 10% of the specular geometry. Right before the measurements, the samples were annealed to 200 K to remove residual CO from the chamber that condensed on the sample during tuning of the electron beam. The recorded spectra exhibited a full width at half-maximum resolution of lower than 35 cm^{-1} (4.4 meV) for the elastic peak in specular geometry.

All calculations were done with the code VASP.[2,3] The interaction between valence and core electrons was modelled with the Projector Augmented Wave (PAW) method,[4,5] while H(1s), C(2s,2p), N(2s,2p), O(2s,2p) and Cu(3d,4s) electrons were treated explicitly with a set of plane waves expanded up to a kinetic energy cutoff of 400 eV. The PBE exchange-correlation functional[6] was adopted, including the long-range dispersion according to the DFT+D2' scheme.[7,8] Structure relaxations were performed with thresholds of 10^{-5} eV and 10^{-2} eV/Å for electronic and ionic loops, respectively. The sampling in the reciprocal space was reduced to the Γ point due to the large dimension of the supercells. The dipole correction was applied along the non-periodic direction and an empty layer of at least 15 Å thickness was included in the supercell. The Cu lattice constant was at first relaxed, yielding 3.57 Å. The Cu(111) surface was then simulated by a five-layers slab, where the ionic coordinates of the three top-most layers were relaxed, while the ions from the two bottom layers were frozen in their bulk positions. A (10 x 10) supercell was adopted, with a lattice parameter of 25.25 Å. Adsorption of a single IPr-NHO molecule was then simulated on this supercell, allowing relaxation of the molecule as well as the substrate (beside the two bottom Cu layers). The adsorption energy, D_e, is defined as the energy of the molecule/substrate adduct with respect to the energy of its separated components:

$$D_e = E(IPrNHO/Cu) - [E(IPrNHO)+E(Cu)] \qquad (1)$$

Negative values of D_e imply stable bonding.

The binding energies of N(1s) core electrons were calculated recurring to the frozen core approximation. The charge transfer between the metal substrate and the adsorbed molecules was evaluated recurring to the Bader model.[9]

The effect of the external electric field is simulated by applying a saw-tooth potential along the non-periodic direction orthogonal to the surface. In Fig. S12 (vide infra) the sign of the electric field is reported with respect to a positive probe charge. It is important to note that the code VASP assumes the opposite sign convention. The harmonic vibrational frequencies were calculated by diagonalizing the mass-weighted Hessian matrix of the second derivatives of the energy with respect to the atomic displacements. Only the adsorbed molecule or dimer and the copper atom directly linked to it were included in the active fragment. In order to keep the computational burden feasible, the thickness of the Cu slab was reduced to two layers in the frequency calculations. The intensities of the calculated normal modes were evaluated by calculating the Born effective charges, i.e. the first derivative of the atomic polarization with respect to the atomic displacements via the Density Functional Perturbation Theory,[10,11] as previously reported in the case of the vibrations of small thiols on transition metal surfaces.[12] The simulated spectra were then generated by fitting a series of Lorentzian functions with a smearing of 3 cm^{-1}.

The comparison with the HREELS spectra was performed by considering specifically the specular or off-specular geometry used in the measurements. In the off specular case, the intensity was calculated from the eigenvector of the atomic displacements and the Born effective charges, as in the dipole approximation:

$$I = \sum_{\alpha}[\sum_{l}\sum_{\beta} Z_{\alpha\beta} e_{\beta}(l)]^2 \quad (2)$$

where $e_{\beta}(l)$ is the eigenvector of the vibrational mode and $Z_{\alpha\beta}$ are the components of the Born effective charge tensor. The sum runs over all l atoms.

In the specular case, we recalculated the intensity by considering only the z-component of the dynamic dipoles:

$$I = \sum_{\alpha}[\sum_{l}\sum_{\beta} Z_{\alpha z} e_{z}(l)]^2 \quad (3)$$

B.- IPr-NHO on Cu(111)

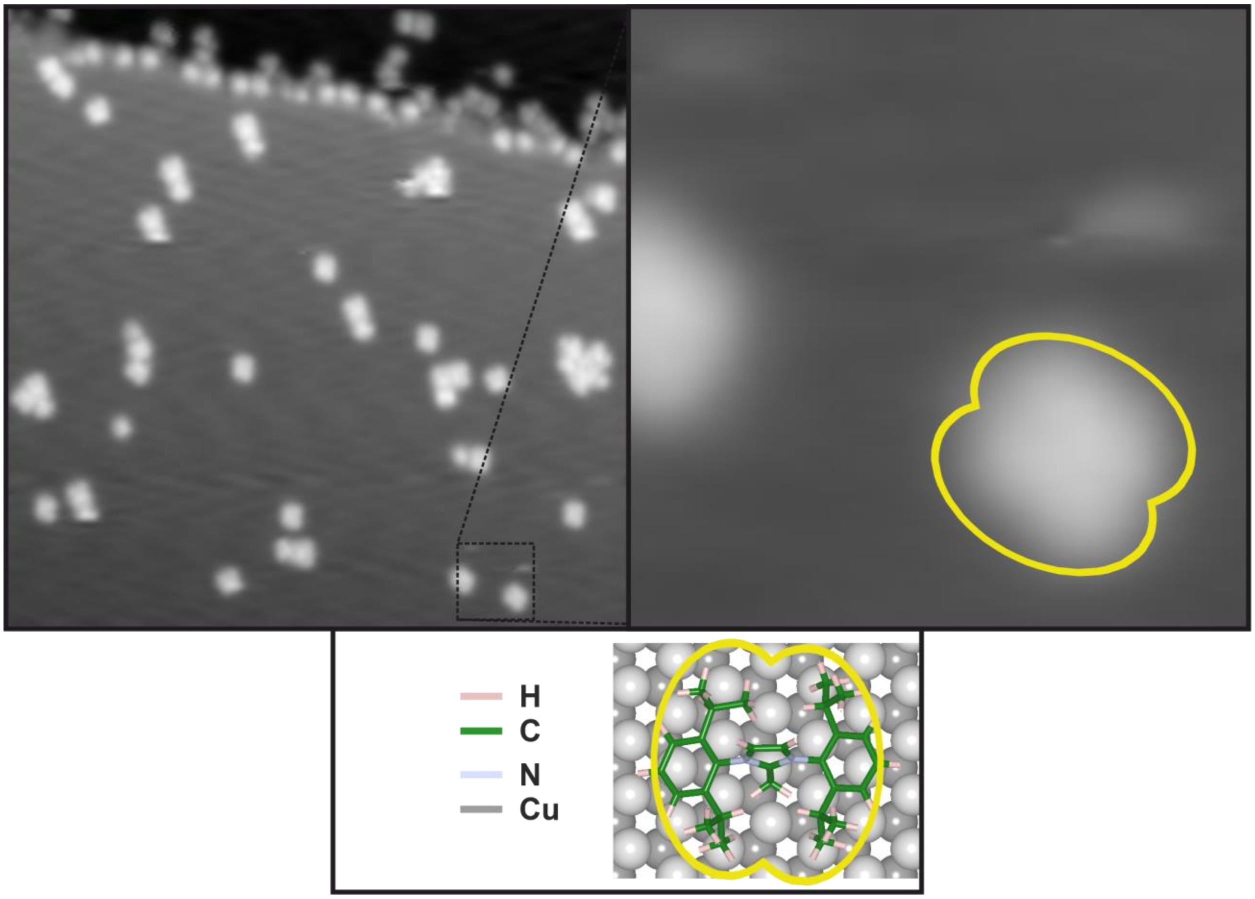

Figure S1. Left top: (40 nm × 40 nm) LT-STM (T = 5K) image of IPr-NHO on Cu(111) IMe-NHC (V_s = -0.8 V, It = 9 pA). Right top: Zoomed in images of IPr-NHO. Bottom: A schematic representation of the binding configuration on the Cu(111) surface.

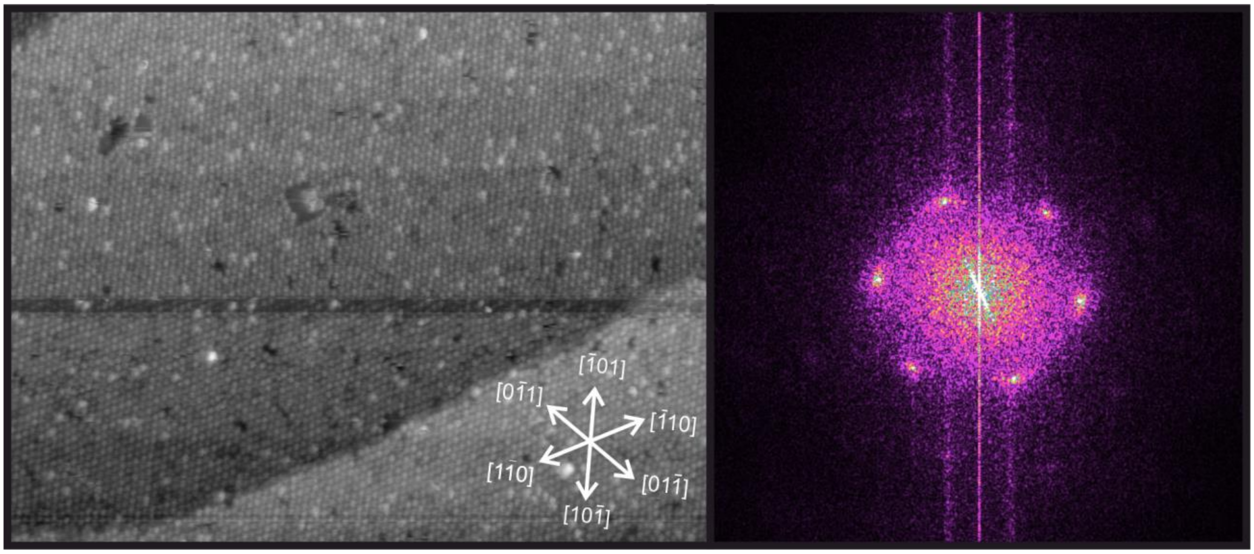

Figure S2. Left: Full coverage of IPr-NHO on Cu(111) (95 nm x 72 nm, V_s = -1.3 V, I_t = 7 pA). The molecules form a tightly packed hexagonal layer aligned with the high symmetry directions of Cu(111) indicated in white. No other rotational domains were observed on other areas of the surface. Right: 2D FFT of the full coverage (5.8 nm^{-1} x 5.8 nm^{-1}).

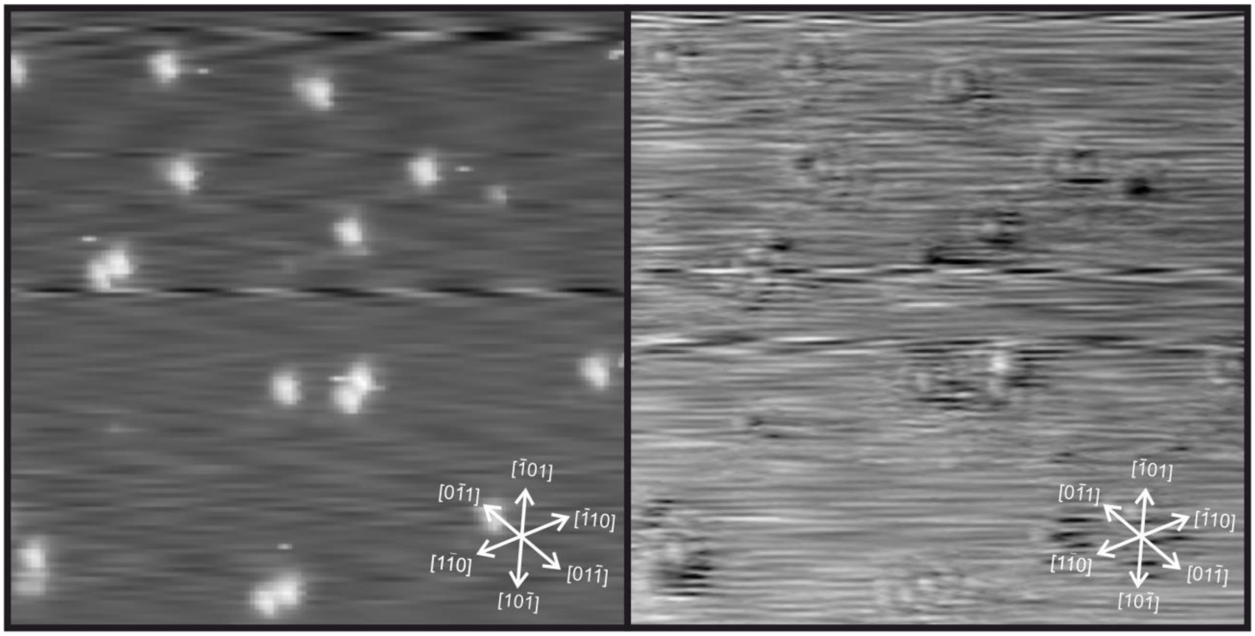

Figure S3. IPr-NHO on Cu(111) measured at negative bias (left, 30 nm x 30 nm, V_s = -1.0 V, I_t = 35 pA) and positive bias (right, 30 nm x 30 nm, V_s = +1.0 V, I_t = 10 pA). The right image shows a high degree of noise with the molecules showing only little contrast. Furthermore, some features around the molecules can be observed that are not seen when measuring at negative bias.

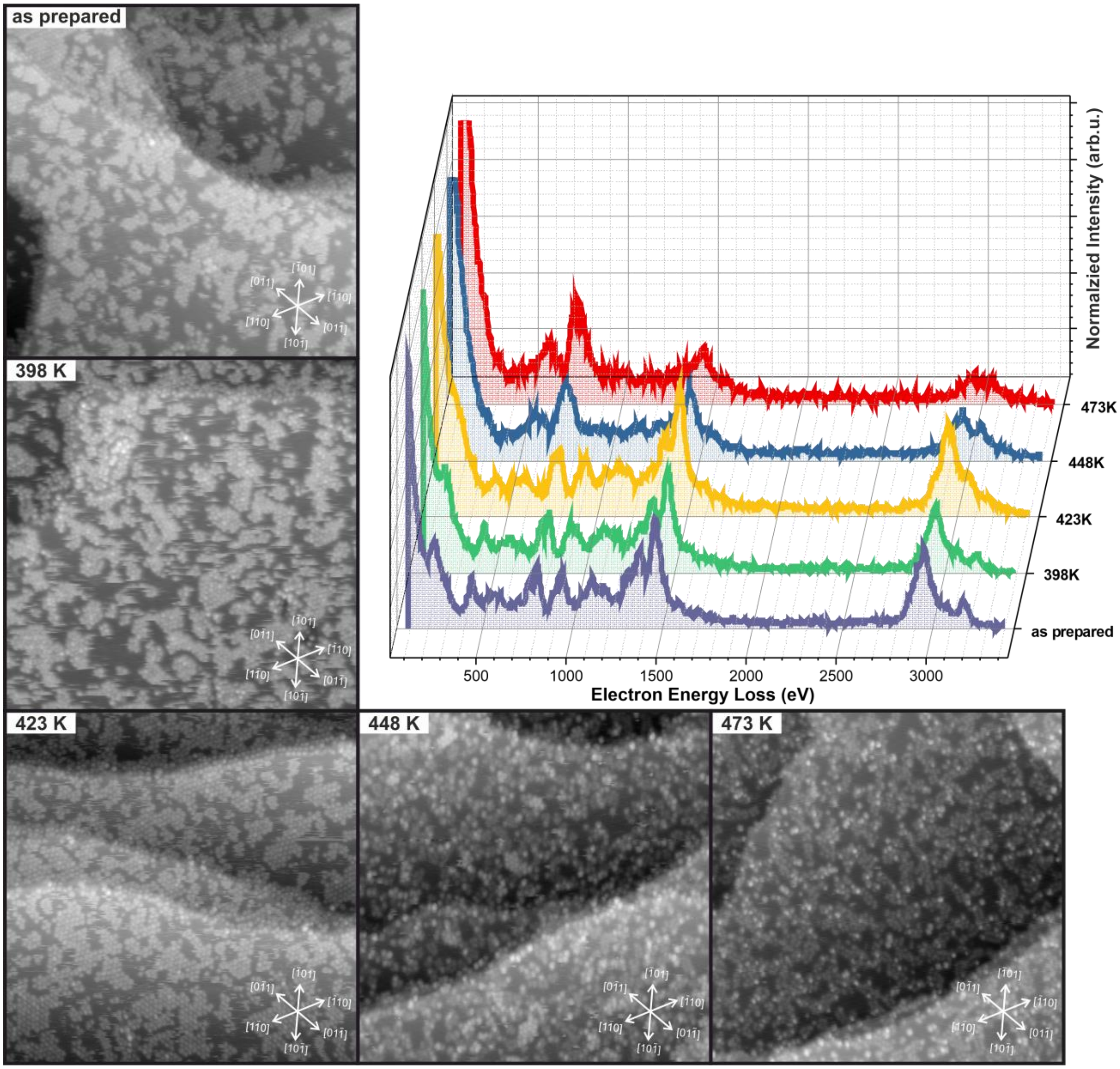

Figure S4. Temperature stability of IPr-NHO on Cu(111) tracked by STM and HREELS. The STM images were measured at 80 nm x 80 nm, V_s = -1.3 V, I_t = 7 pA. The HREELS spectra were measured in specular scattering geometry, with the FWHM of the main peak kept below 30 cm^{-1}: The molecules are unchanged up to 398 K. At 448 K the molecular arrangement starts to deteriorate and individual molecules become less clearly discernible. This is accompanied by a reduction of discernible vibrational modes, indicating a molecular decomposition. The STM images were taken at different areas for each annealing step.

C.- “Writing” Process of IPr-NHO on Cu(111)

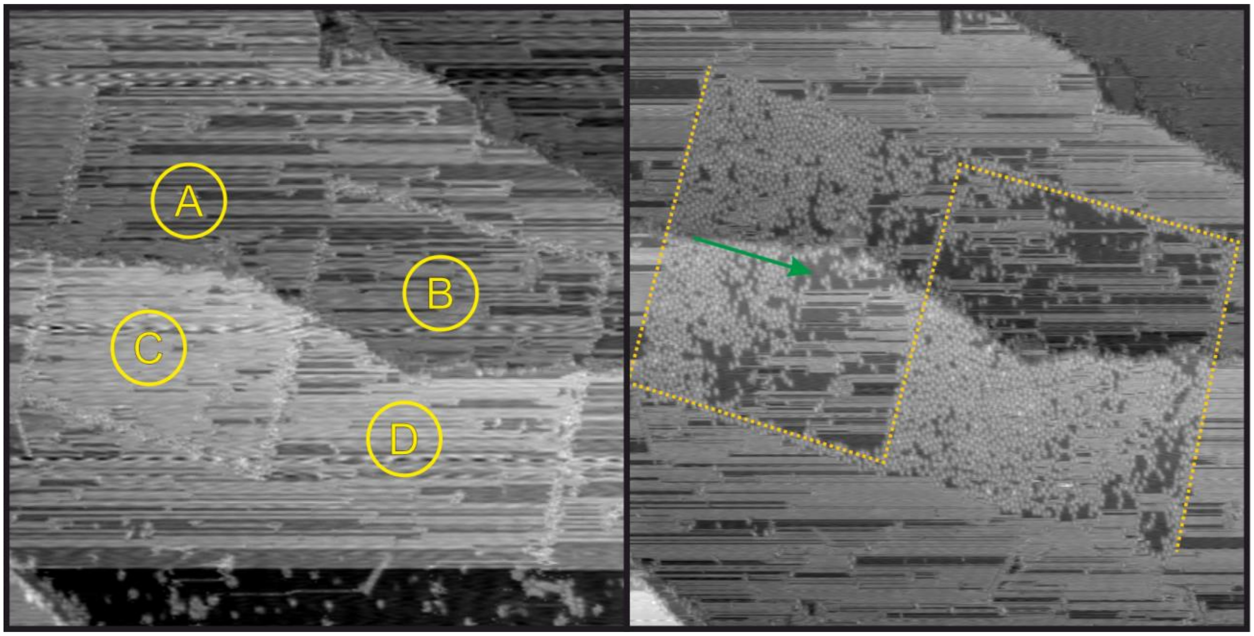

Figure S5. STM images tracking the “writing” of IPr-NHO/Cu(111). All images were measured at 140 nm x 140 nm, V_s = -0.9 V, I_t = 9 pA. Similar to Figure S4, a barrier was drawn (left) creating four different areas: open, lower terrace (A), closed, lower terrace (B), closed, upper terrace (C) and open, upper terrace (D). Subsequently, an image was taken (right) after a rectangular scanning image at “writing” conditions (120 nm x 70 nm, V_s = +2.5 V, I_t = 150 pA) was taken in the direction marked in green (right). While A and D are almost completely filled with additional molecules, B and C are only partially filled, indicating the physisorbed species prior to “writing” cannot move across terrace steps or bound molecules.

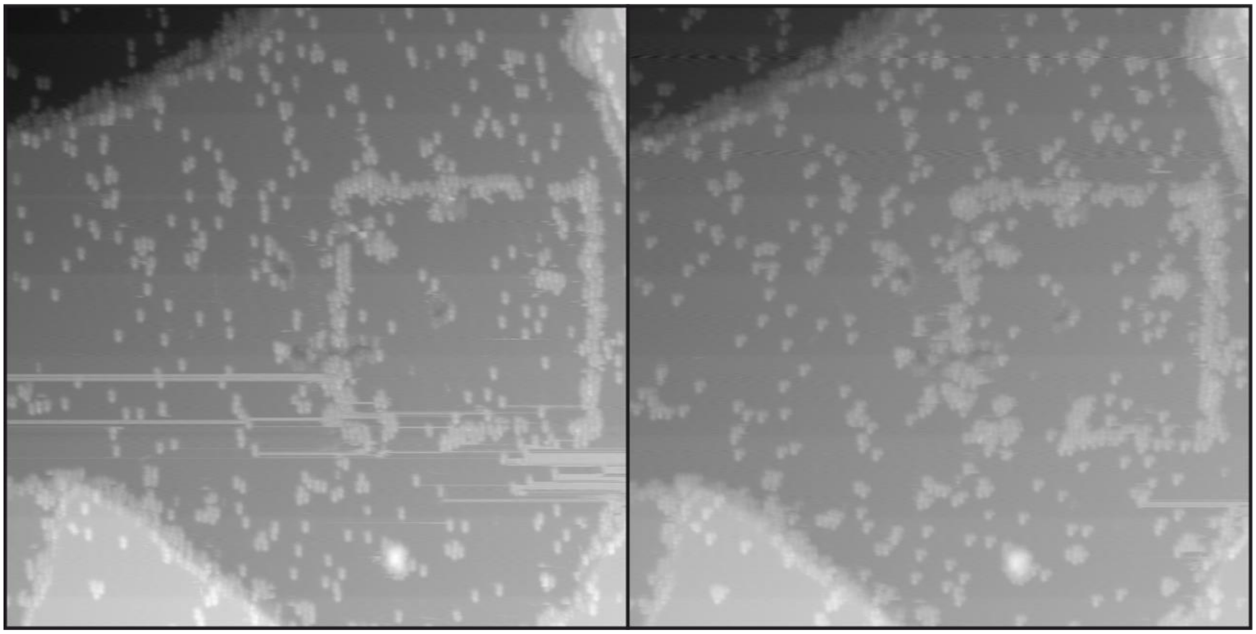

Figure S6. LT-STM image of IPr-NHO on Cu(111) acquired after "writing" a square by moving the STM tip over the surface. The image on the left was taken immediately after the writing attempt, the image on the right was taken after 10.5 h at continuous measurements (230 nm x 230 nm, V_s = -0.8 V, I_t = 10 pA).

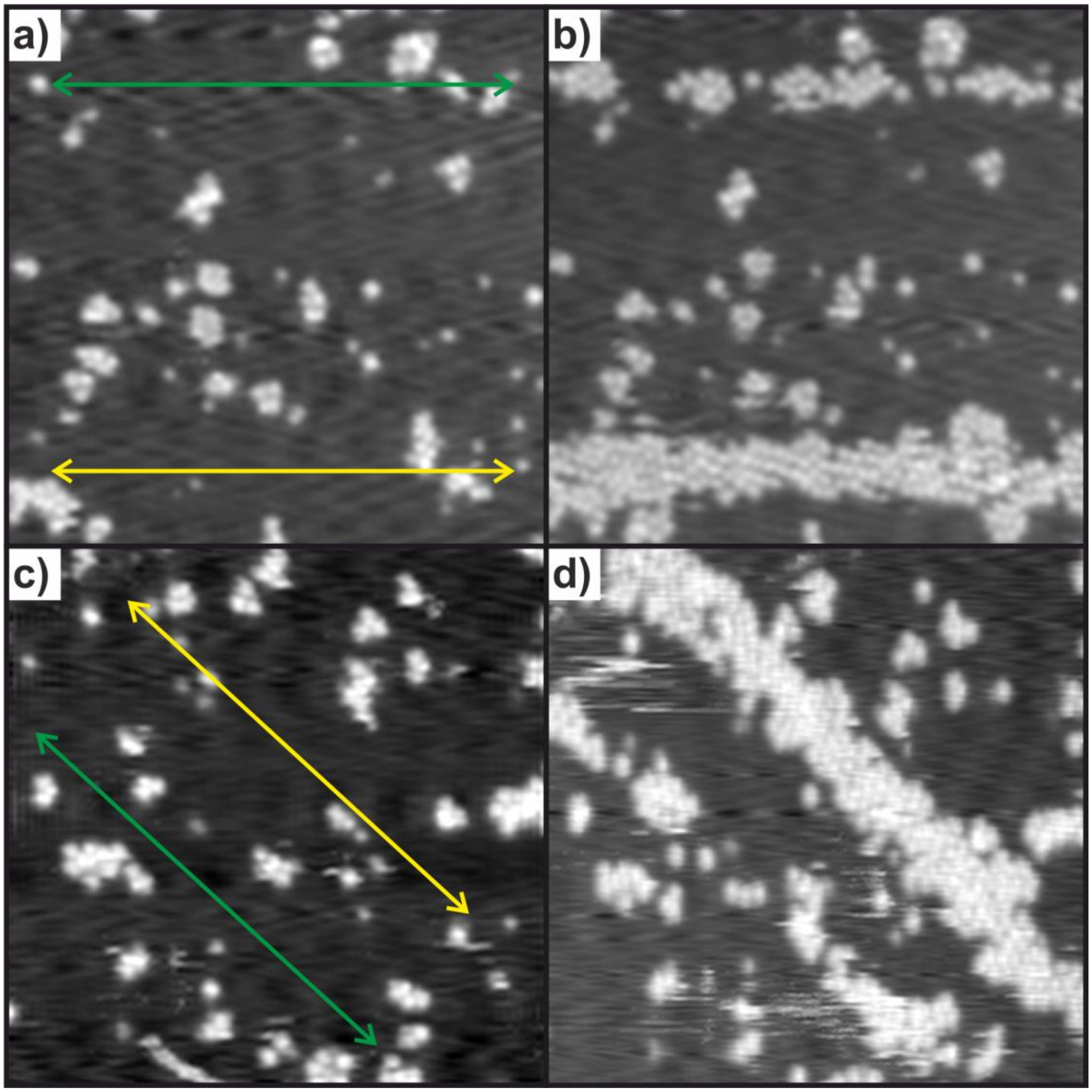

Figure S7. Influence of different tunneling conditions on the "writing" process. a,c) Two lines were "written" by positioning the tip over the surface, switching to writing conditions (+2.5 V) and dragging the tip in a line across the surface and back at 10 nm/s with a current of 8 pA (green) and 250 pA (yellow). In a) the line indicated in green was drawn first, in c) the line indicated in yellow was drawn first. b,d) images measured after "writing" two lines, respectively. All images: 45 nm x 45 nm, V_s = -0.9 V, I_t = 8 pA. The tip-sample distance corresponds to the measured tunnelling current with higher currents being associated with lower tip-sample distances.

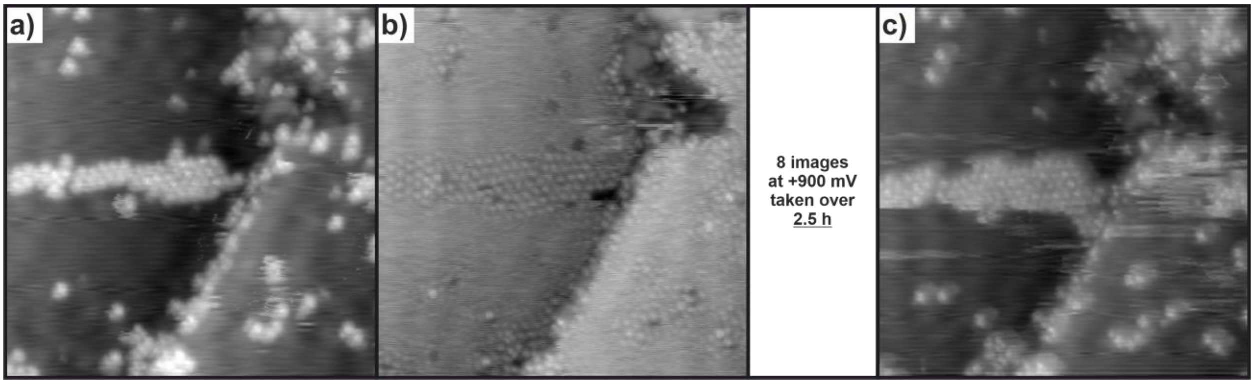

Figure S8. Scanning at low positive bias voltages. a) (40 nm x 40 nm, V_s = -0.9 V, I_t = 8 pA) images scanned first. b) (40 nm x 40 nm, V_s = +0.9 V, I_t = 8 pA) image scanned subsequently at low positive bias shows a noisy background. Additional molecules appear around previously observed molecules, some of them seem very faint against the noisy background. Multiple images were scanned at these conditions for 2.5 h until c) (40 nm x 40 nm, V_s = -0.9 V, I_t = 8 pA) was taken. Compared to a), a few additional molecules have appeared at previously present patches.

D.- IPr-NHO on Cu(100)

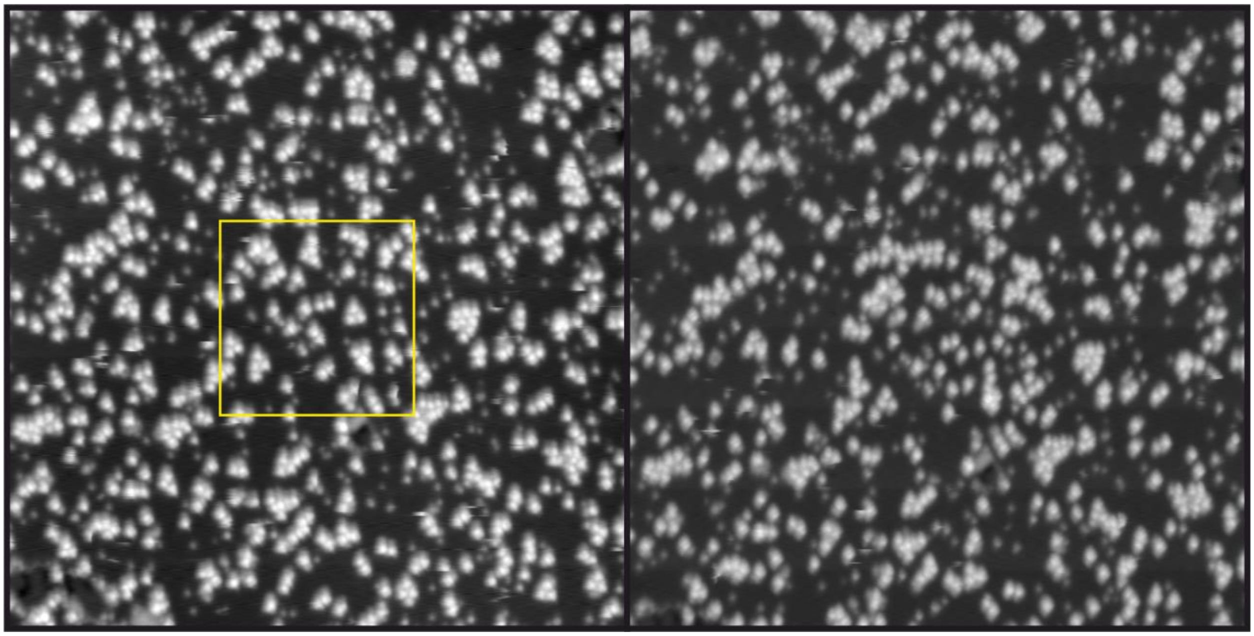

Figure S9. LT-STM images of IPr-NHO on Cu(100) before (left) and after (right) an attempt to "write" molecules in the are indicated in yellow. Writing conditions: 25 nm x 25 nm, V_s = +2.4 V, I_t = 70 pA. Both images were measured at 80 nm x 80 nm, V_s = -1.3 V, I_t = 7 pA.

E.- XPS

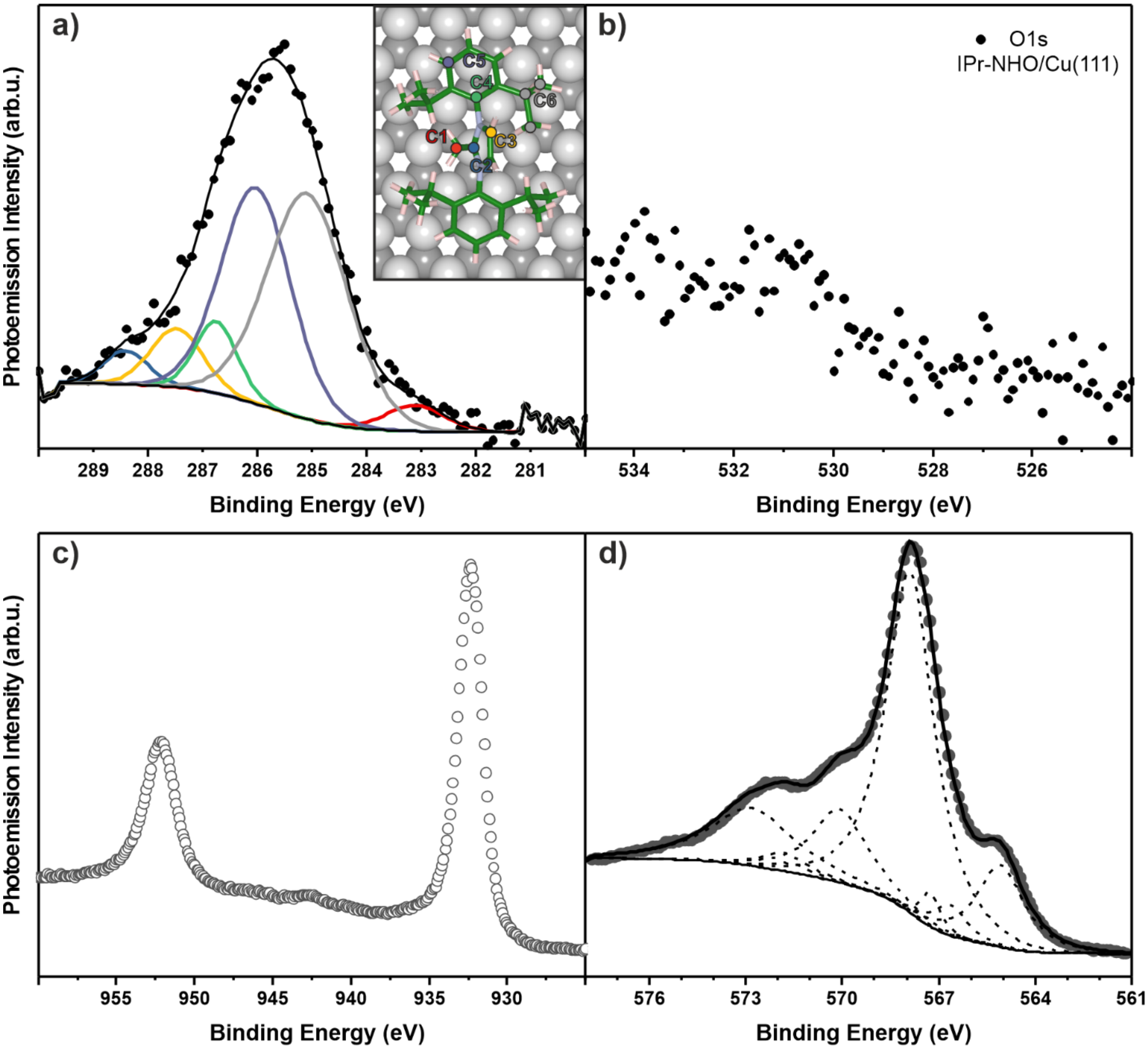

Figure S10. XPS data of IPr-NHO on Cu(111). a) C 1s spectra. The components of the chemically discernible C atoms has been fitted reflecting the stoichiometric ratios 1:1:2:2:10:12. The large FWHM of C1 reflects the presence of physisorbed and chemisorbed species on the surface. b) O1s spectra showing no significant signal, indicating successful decarboxylation of the CO_2-adduct precursor. c) Cu 2p spectra. d) Cu $L_3M_{45}M_{45}$ Auger spectra which has been fitted in accordance with reported analysis.[13]

F.- Calculated Structures HREELS Vibrational assignment

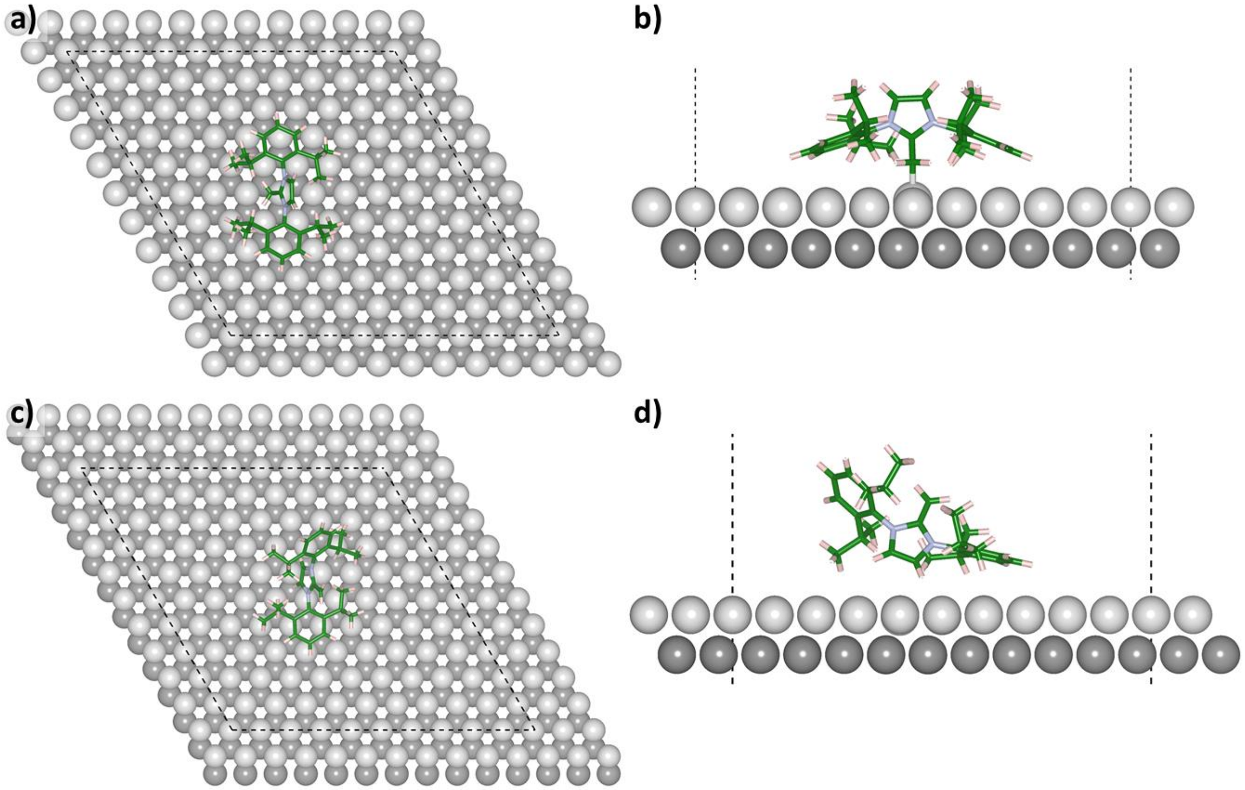

Figure S11. (a, b) Top and side view of chemisorbed (Cu-CH_2) IPr-NHO, (c, d) Top and side view of physisorbed IPr-NHO.

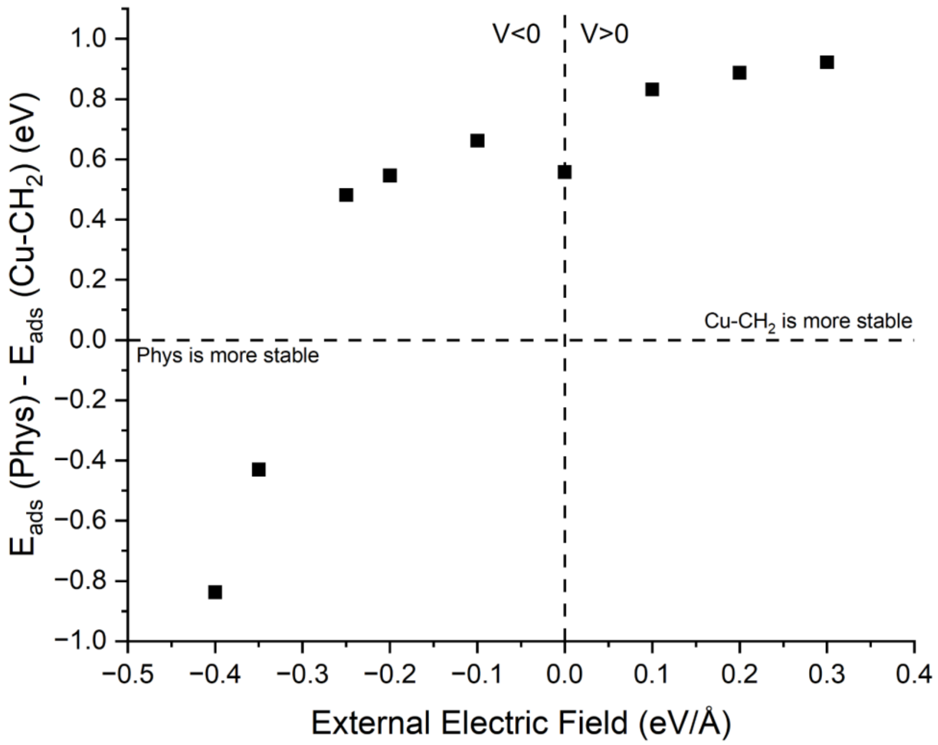

Figure S12. Effect of the external electric field on the relative stability of chemisorbed (Cu-CH_2) and physisorbed IPr-NHO. Positive electric field corresponds to positive bias voltage (see Figure 6).

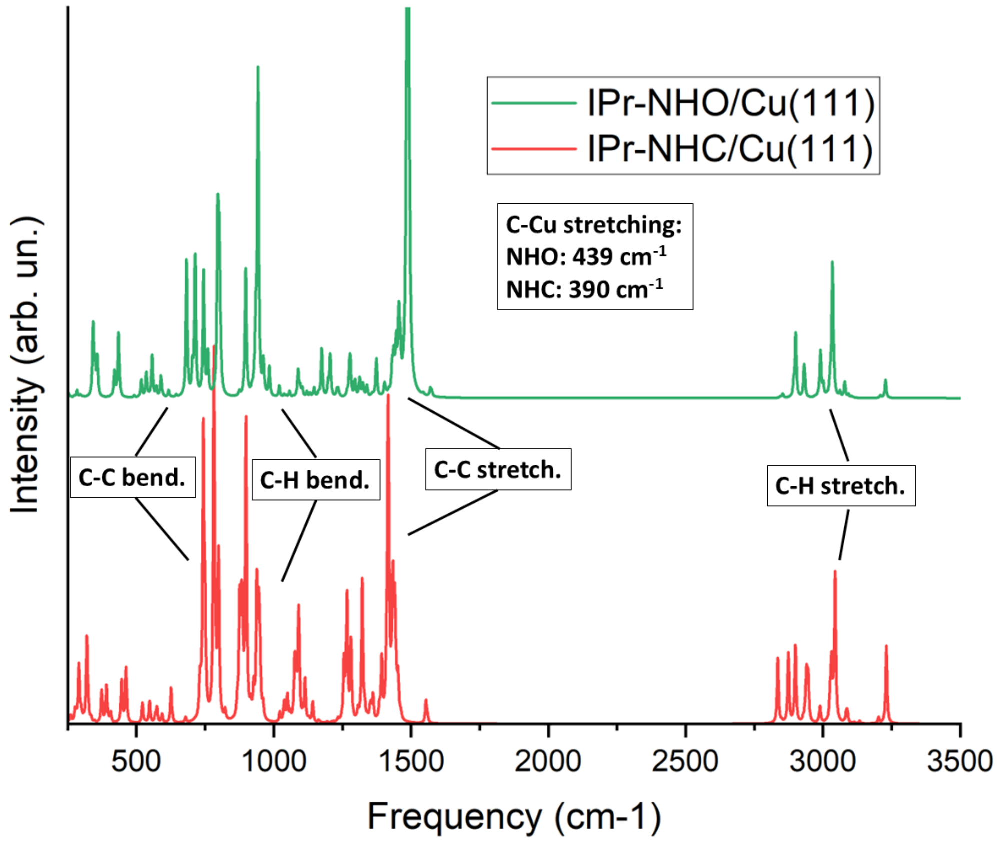

Figure S13. Calculated HREELS spectra of IPr-NHO and IPr-NHC on Cu(111) and related assignment of the most important normal modes.

The C-Cu stretching mode falls at 390 cm^{-1} (IPr-NHC) and 440 cm^{-1} (IPr-NHO). The C-C and C-H bending modes are responsible for several multiplets in the 750-1500 cm^{-1} region. The C-H bending modes in IPr-NHC have a stronger signal around 1250 cm^{-1} compared to IPr-NHO, due to the more vertical arrangement of the molecule. Noteworthy, the C-C stretching frequency region of IPr-NHO is extended up to higher frequencies compared to NHC.

G.- References

1 M. Das, C. Hogan, R. Zielinski, M. Kubicki, M. Koy, C. Kosbab, S. Brozzesi, A. Das, M. T. Nehring, V. Balfanz, J. Brühne, M. Dähne, M. Franz, N. Esser and F. Glorius, N-Heterocyclic Olefins on a Silicon Surface, *Angew. Chemie Int. Ed.*, 2023, **62**, e202314663.

2 G. Kresse and J. Furthmüller, Efficient iterative schemes for ab initio total-energy calculations using a plane-wave basis set, *Phys. Rev. B*, 1996, **54**, 11169–11186.

3 G. Kresse and J. Furthmüller, Efficiency of ab-initio total energy calculations for metals and semiconductors using a plane-wave basis set, *Comput. Mater. Sci.*, 1996, **6**, 15–50.

4 P. E. Blöchl, Projector augmented-wave method, *Phys. Rev. B*, 1994, **50**, 17953–17979.

5 G. Kresse and D. Joubert, From ultrasoft pseudopotentials to the projector augmented-wave method, *Phys. Rev. B*, 1999, **59**, 1758–1775.

6 J. P. Perdew, K. Burke and M. Ernzerhof, Generalized Gradient Approximation Made Simple, *Phys. Rev. Lett.*, 1996, **77**, 3865–3868.

7 S. Grimme, Semiempirical GGA-type density functional constructed with a long-range dispersion correction, *J. Comput. Chem.*, 2006, **27**, 1787–1799.

8 S. Tosoni and J. Sauer, Accurate quantum chemical energies for the interaction of hydrocarbons with oxide surfaces: CH4/MgO(001), *Phys. Chem. Chem. Phys.*, 2010, **12**, 14330.

9 R. F. W. Bader, A Quantum Theory of Molecular Structure and Its Applications, *Chem. Rev.*, 1991, **91**, 893–928.

10 M. Gajdoš, K. Hummer, G. Kresse, J. Furthmüller and F. Bechstedt, Linear optical properties in the projector-augmented wave methodology, *Phys. Rev. B*, 2006, **73**, 045112.

11 S. Baroni and R. Resta, Ab initio calculation of the macroscopic dielectric constant in silicon, *Phys. Rev. B*, 1986, **33**, 7017–7021.

12 D. Karhánek, T. Bučko and J. Hafner, A density-functional study of the adsorption of methane-thiol on the (111) surfaces of the Ni-group metals: II. Vibrational spectroscopy, *J. Phys. Condens. Matter*, 2010, **22**, 265006.

13 N. Pauly, S. Tougaard and F. Yubero, LMM Auger primary excitation spectra of copper, *Surf. Sci.*, 2014, **630**, 294–299.
